# Mammography Screening in a Large Health System Following the U.S. Preventive Services Task Force Recommendations and the Affordable Care Act

**DOI:** 10.1371/journal.pone.0131903

**Published:** 2015-06-29

**Authors:** Heidi D. Nelson, Roshanthi Weerasinghe, Lian Wang, Gary Grunkemeier

**Affiliations:** 1 Providence Cancer Center, Providence Health and Services, Portland, Oregon, United States of America; 2 Department of Medical Informatics and Clinical Epidemiology, Oregon Health and Science University, Portland, Oregon, United States of America; 3 Department of Medicine, Oregon Health and Science University, Portland, Oregon, United States of America; 4 Medical Data Research Center, Providence Health & Services, Portland, Oregon, United States of America; ISPO, ITALY

## Abstract

**Background:**

Practice recommendations for mammography screening were issued by the U.S. Preventive Services Task Force in 2009 and expansion of insurance coverage was provided under the Patient Protection and Affordable Care Act soon thereafter, yet the influence of these changes on screening practices in the United States is not known.

**Methods:**

To determine changes in mammography screening and their associations with new practice recommendations and the Affordable Care Act, we examined patient-level data from 249,803 screening mammograms from January 1, 2008 through December 31, 2012 in a large community-based health system in the northwestern United States. Associations were determined by an intervention analysis of time-series data method.

**Results:**

Among women screened, 64% were age 50-74 years; 84% self-identified as white race; 62% had commercial insurance; and 70% were seen in facilities located in metropolitan areas. Practice recommendations were associated with decreased screening volumes among women age <40 (-37.4 mammograms/month; -39.4% change; *P*<0.001), 40-49 (-106.0 mammograms/month; -11.2% change; *P*<0.001), and ≥75 (-54.7 mammograms/month; -10.0% change; *P*<0.001), but not women age 50-74. Implementation of the Affordable Care Act was associated with increased screening among women age 50-74 (+184.3 mammograms/month; +7.2% change; *P*=0.001), but not women <40 or ≥75; increases for age 40-49 were of borderline statistical significance (+56.9 mammograms/month; +6% change; *P*=0.06). Practice recommendations were also associated with decreased screening for women with commercial insurance, while the Affordable Care Act was associated with increased screening for women with Medicare, Medicaid, or other noncommercial sources of payment.

**Conclusions:**

Mammography screening volumes in a large community health system decreased among women age <50 and ≥75 in association with new U.S. Preventive Services Task Force practice recommendations, while insurance coverage changes under the Affordable Care Act were associated with increased screening volumes among women age 50-74.

## Introduction

In November 2009, the U.S. Preventive Services Task Force (USPSTF) issued new age-based recommendations for mammography screening for women without high risks for breast cancer [1] based on research of its benefits and harms [2]. For all women age 50–74 years, they recommended routine mammography every two years. For women age 40–49, they recommended selective screening depending on risk factors for breast cancer and personal values regarding the trade-offs between benefits and harms. The USPSTF acknowledged that research among women age 75 and older was too limited to support a recommendation and screening would depend on individual considerations. The new recommendations represented a major change from the previous practice of screening every one to two years beginning at age 40 [3] that continues to be supported by several organizations in the United States [4–6].

Four months later, the Patient Protection and Affordable Care Act was passed by the U.S. Congress mandating insurance coverage for mammography screening with no co-pay or deductible fees [7]. Coverage includes yearly mammography for women age 40 and older beginning in the next plan year after October 2010 for health plans created after passage of the law. Although Medicare, Medicaid, and all but one state had existing regulations regarding coverage of mammography screening, these varied widely [8].

How these changes influenced breast cancer screening practices among women in the United States is unclear. Studies of the effects of the new USPSTF recommendations have mixed results. Surveys of women providing self-reported mammography information indicated no statistically significant changes in screening rates [9, 10], whereas data from a state-wide registry of screening mammography [11] and from insurance claims [12] showed significant decreases in screening. These discrepancies may be due to the methodological limitations of self-reported survey data that have been shown to overestimate actual mammography rates [13,14], although other contributing factors may also exist. A study of women in their forties indicated a modest decline in screening rates initially after the new guidelines were released, followed by an increase in rates two years later [15]. However, this study did not consider the subsequent effects of the Affordable Care Act on mammography screening. Understanding the influence of both of these changes is important to consumers, policy makers, and health systems responsible for delivering effective screening services.

The objective of this study is to consider the influences of both the new USPSTF recommendations and the implementation of the Affordable Care Act on changes in mammography screening by examining patient-level mammography data across five years from women receiving care in a large community-based health system.

## Methods

This study was approved by the Providence Institutional Review Board and Providence Privacy Board. Informed consent was not required because the data were analyzed anonymously. This study is an intervention analysis of patient-level time-series data obtained from January 1, 2008 through December 31, 2012. Monthly mammography screening volume is the main outcome measure.

### Data Sources

The study was based at a large not-for-profit community health system serving urban and rural populations in Oregon and southwest Washington (Providence Health & Services, Oregon Region). This is an integrated health system of eight community hospitals and affiliated outpatient facilities that provides comprehensive care for breast cancer and related conditions including screening, diagnosis, treatment, and survivorship care. Since Providence is an open-access health system, patients are covered by a variety of private and public health insurance plans or receive unsponsored or charity care, and closely match the demographic and socio-economic profiles of their communities. Physician practices are similarly varied, and include private as well as health system employed groups. While the health system provided guidance about mammography screening on their website that incorporated much of the language and rationale of the USPSTF recommendation, it issued no specific requirements or mandates [16].

Data for this analysis were obtained from women receiving mammography screening within the health system as part of their usual health care. The health system uses a master patient identifier for each patient accessing its extensive clinical network of hospitals and clinics. The master patient identifier and other unique patient identifiers are used to track patients across multiple encounters and over time. A breast care specific data mart developed by the health system integrates patient-level data from various internal sources, including administrative databases, electronic medical records, imaging data, and pathology data from the laboratory information system [17, 18]. Data are subsequently linked based on matching algorithms that group data for individual patients creating disease-specific data tables accessed by customized interactive queries. Data security and patient confidentiality are protected by existing health system safeguards and procedures.

All screening mammograms (Current Procedural Terminology codes G0202/77052) obtained within the health system from January 1, 2008 through December 31, 2012 were imported to the data mart. Mammograms obtained for purposes other than screening, such as diagnostic or unilateral views, were not included. Each patient provided data for only one screening mammogram per year. The study dates were selected to capture mammography volume before, during, and after the release of the USPSTF screening recommendations in November 2009 and implementation of coverage for mammography screening under the Affordable Care Act in January 2011. Although coverage was implemented by different health plans at different times, January 1, 2011 represents the point in which the majority of plans at the health system had changed their coverage policies for mammography screening because it was the start of a new insurance year.

Although our primary patient variable was age at the time of mammography, we were also interested in how other factors influenced screening changes, including insurance type, race and ethnicity (self-reported by patients), and location of mammography. Thus, each patient’s medical record number, date of birth, date of mammography, race and ethnicity, insurance type, and facility location were also imported to the data mart.

### Statistical Analysis

Age was categorized according to age-based screening recommendations (<40, 40–49, 50–74, and ≥75 years); insurance type by commercial, Medicare, Medicaid, and other sources of payment; and facility location by whether it was located in a metropolitan or non-metropolitan area. Comparisons of age, insurance type, and facility location by year; and age by insurance type and facility location were determined by the Chi-square test.

Monthly mammography volumes across the health system were treated as time series data that could be affected by the two specified interventions: the announcement of new USPSTF screening recommendations and the implementation of insurance coverage under the Affordable Care Act. We used this approach because it provides a method to assess the effect of interventions over time despite the influence of multiple other effects that are not related to the primary interventions, such as background noise and seasonal changes, and in the absence of a fixed patient denominator.

Using an intervention analysis for time series data method [19], we modeled monthly mammography volume by simultaneously incorporating two additive parts. First, the underlying intrinsic fluctuation (i.e. fluctuations attributed to background noise and seasonal changes) was modeled using the integrated autoregressive moving average (ARIMA) regression approach. Secondly, the effects of interventions (i.e., new recommendations and insurance coverage) on the mean monthly mammography volume were modeled as simple step changes. For this study, the interventions were presented as step functions at the time of the start of their implementation (December 2009 for USPSTF recommendations and January 2011 for the Affordable Care Act), and their effects were assumed to be simple step changes in the screening process. Mean monthly mammography volumes were obtained from the model for the effect of no intervention to establish baseline measures and for the effect of change due to each intervention. The analysis was repeated stratified by age (<40, 40–49, 50–74, and ≥75 years) and insurance categories (commercial, Medicare, Medicaid, and other sources of payment). To account for the possibility that changes occurred in a graded rather than step fashion, we modified the modeling process with a ramping up effect to see if results differed. All analyses were performed using R software (version 3.1.0); the intervention analysis used the TSA package [20].

## Results

A total of 249,803 screening mammograms were obtained from January 1, 2008 through December 31, 2012. The majority of mammograms were from women between the ages of 50–74 years (64.1%); and 21.8% were 40–49, 12.5% were 75 and older, and 1.7% were younger than 40 (Table 1).

**Table 1:** Screening Mammograms by Age, n (row %).

		Age, y			
Characteristic	Total	<40	40–49	50–74	≥75
**Total**	249,803	4,119 (1.7)	54,342 (21.8)	160,035 (64.1)	31,307 (12.5)
**Year**					
2008	50,298	1,226 (2.4)	11,934 (23.7)	30,427 (60.5)	6,711 (13.3)
2009	50,063	995 (2.0)	11,179 (22.3)	31,136 (62.2)	6,753 (13.5)
2010	48,713	686 (1.4)	10,173 (20.9)	31,633 (64.9)	6,221 (12.8)
2011	51,693	660 (1.3)	10,965 (21.2)	33,940 (65.7)	6,128 (11.9)
2012	49,036	552 (1.1)	10,091 (20.6)	32,899 (67.1)	5,494 (11.2)
**Insurance type**					
Commercial	154,886	3,749 (2.4)	48,636 (31.4)	102,100 (65.9)	401 (0.3)
Medicare	81,189	53 (0.1)	1,269 (1.6)	49,004 (60.4)	30,863 (38.0)
Medicaid	5,678	194 (3.4)	2,051 (36.1)	3,416 (60.2)	17 (0.3)
Other sources^a^	8,050	123 (1.5)	2,386 (29.6)	5,515 (68.5)	26 (0.3)
**Facility location**					
Metro	175,407	3,132 (1.8)	39,810 (22.7)	112,123 (63.9)	20,342 (11.6)
Non-metro	74,396	987 (1.3)	14,532 (19.5)	47,912 (64.4)	10,965 (14.7)

^a^Other sources include self-pay and charity care.

At baseline, the types of insurance coverage varied across age groups (*P*<0.001). Most women age 74 and younger were covered by commercial insurance (61.9%), while nearly all women age 75 and older were covered by Medicare (98.6%). Overall, 2.3% of women were covered by Medicaid and 3.2% by other sources including self-pay and charity care. When adjusted for age, the proportion of women covered by commercial insurance decreased over time (65.1% in 2008 to 60.3% in 2012, *P*<0.001), while proportions covered by Medicare, Medicaid, or other sources increased.

The majority of women in all age groups obtained their mammography in health system facilities located in a metropolitan compared with non-metropolitan area (70.2% versus 29.8%, *P*<0.001). This distribution did not change over time and was not further considered in the statistical models. The majority of women were white (84%), followed by a category including combined, other, and unknown racial heritage (8%), Asian (6%), and African American (2%). The completeness of data on race and ethnicity varied between facility locations, rendering it unreliable for statistical models.

### Associations of the USPSTF Recommendations and the Affordable Care Act on Screening Volume

Mammography screening volume (mean number of mammograms/month) displayed yearly cyclic changes. These cycles have been observed over preceding years and are related to outreach activities during breast cancer awareness month each October as well as general increases in health care utilization at the end of each insurance year. Overall, the USPSTF recommendations were associated with a decrease in volume that was not statistically significant (-138.9 mammograms/month, *P* = 0.10), while implementation of the Affordable Care Act was associated with increased volume (+232.7 mammograms/month, *P* = 0.006) (Table 2).

**Table 2:** Associations of Screening Recommendations and Affordable Care Act Implementation on Volume of Screening Mammography.

		USPSTF Screening Recommendations				Affordable Care Act Implementation			
Characteristic	Baseline^a^ Mean (mam/mo)	Mean Change (mam/mo)	SE	*P*-value	% Change	Mean Change (mam/mo)	SE	*P*-value	% Change
**Overall**	4146.9	-138.9	84.9	0.10	-3.3	232.7	85.0	0.006	5.6
**Age, *y***									
<40	94.9	-37.4	6.6	<0.001	-39.4	-6.0	6.5	0.36	-1.1
40–49	948.2	-106.0	30.4	<0.001	-11.2	56.9	30.2	0.06	6.0
50–74	2586.8	7.7	57.0	0.89	0.3	184.3	56.5	0.001	7.2
≥75	549.2	-54.7	15.7	<0.001	-10.0	-6.6	16.0	0.68	-1.2
**Insurance type**									
Commercial	2659.6	-125.5	52.4	0.02	-4.7	31.5	52.4	0.55	1.2
Medicare	1342.5	-30.5	16.1	0.06	-2.3	86.2	15.5	<0.001	6.4
Medicaid	83.7	4.7	6.9	0.50	5.6	20.5	6.5	0.002	24.5
Other sources	96.6	23.6	14.5	0.10	24.4	43.3	14.5	0.003	44.8

^a^2008 baseline estimation from each model. Although the sub-groups do not sum to the overall baseline estimate numerically, they are essentially the same when the uncertainty (SE) of the estimation is taken into account.

**Fig 1:**
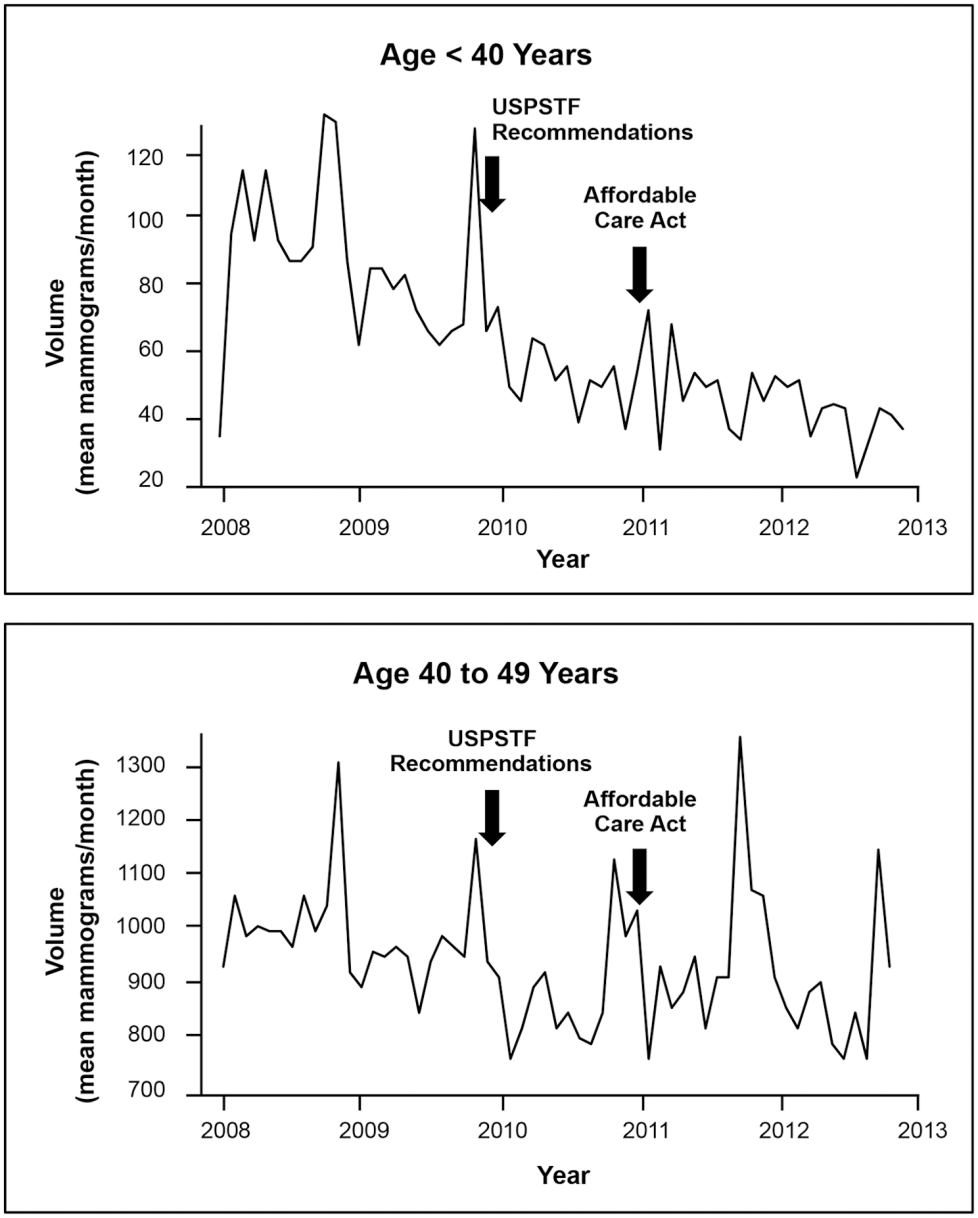
Screening Mammography Changes for Women <40 and 40–49. The mean number of screening mammograms per month performed in the health system from 2008 through 2012. Arrows indicate the times of new screening recommendations and implementation of the Affordable Care Act. Recommendations were associated with decreased screening for women <50, while the Affordable Care Act had no statistically significant associations.

**Fig 2:**
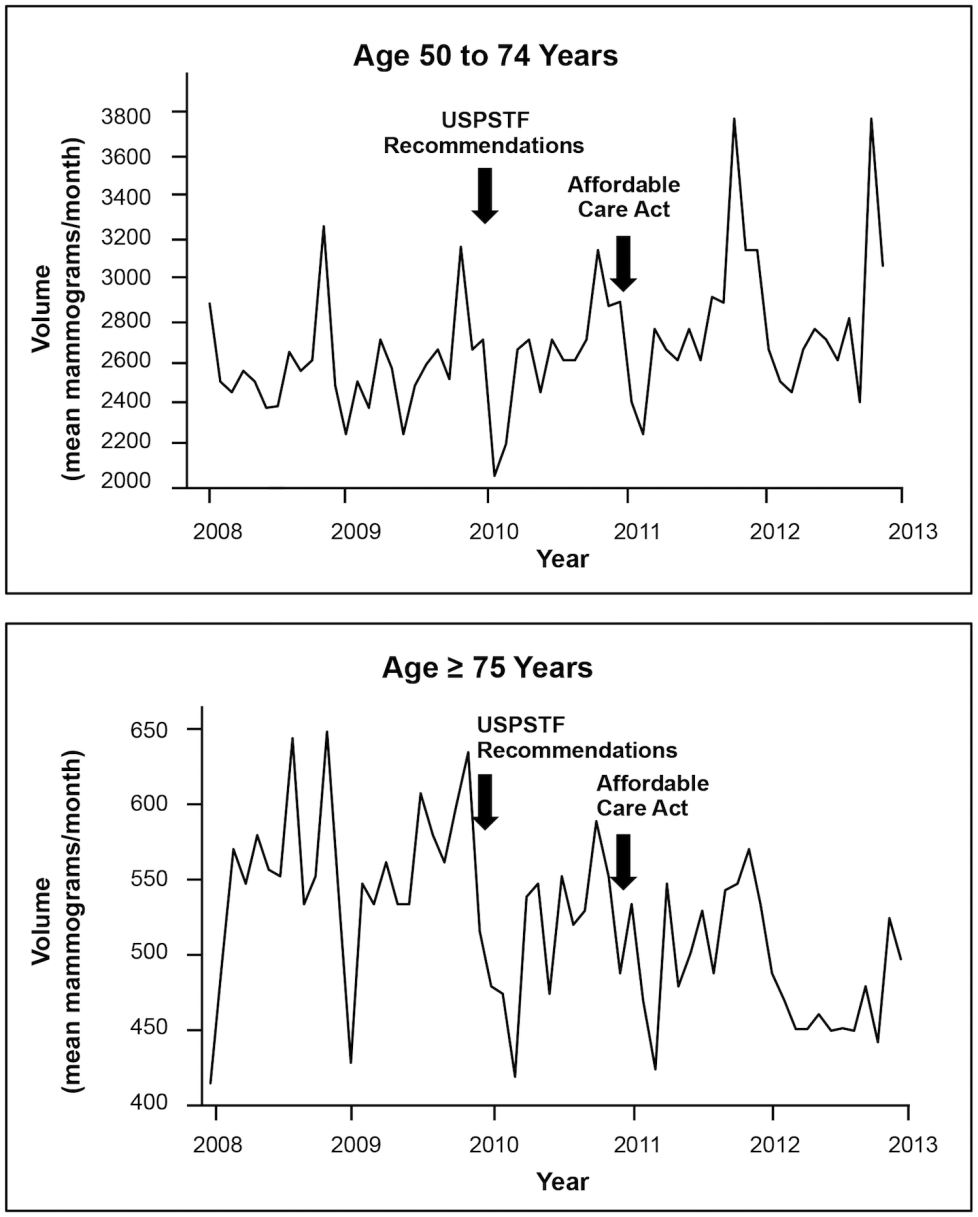
Screening Mammography Changes for Women 50–74 and ≥75. The mean number of screening mammograms per month performed in the health system from 2008 through 2012. Arrows indicate the times of new screening recommendations and implementation of the Affordable Care Act. New recommendations were not associated with changes for women age 50–74, but were associated with decreased screening for ≥75, while the Affordable Care Act was associated with increased screening among women age 50–74 and no changes for ≥75.

The effect of the USPSTF recommendations on screening volume varied by age and insurance type. Volumes decreased from baseline for women younger than 40 (-37.4 mammograms/month, *P*<0.001), 40–49 (-106.0 mammograms/month, *P*<0.001) (Fig 1), and 75 and older (-54.7 mammograms/month, *P*<0.001), but did not change for women 50–74 (Fig 2). When stratified by insurance type, volume decreased from baseline for commercial insurance (-125.5 mammograms/month, *P* = 0.02), but not for other insurance types.

Implementation of the Affordable Care Act was associated with increased screening volume for women 50–74 (+184.3 mammograms/month, *P* = 0.001), and among women with Medicare (+86.2 mammograms/month, *P*<0.001), Medicaid (+20.5 mammograms/month, *P* = 0.002), and other noncommercial sources of payment (+43.3 mammograms/month, *P* = 0.003) compared to baseline (Table 2). Changes were not statistically significant in other age groups or for commercial insurance.

Results were similar when we modified the modeling process with a ramping up effect to account for gradual rather than stepped interventions.

## Discussion and Conclusions

Mammography screening in a community health system increased among women age 50–74 and decreased for other ages over five years in association with new practice recommendations and insurance coverage changes. While the USPSTF recommendations were specifically associated with decreased screening among women younger than 50 and 75 and older, but not women age 50–74, the Affordable Care Act was associated with increased screening among women age 50–74, but not women in the other age groups.

These results are consistent with current practice standards and quality performance measures, such as revised HEDIS (Healthcare Effectiveness Data and Information Set) measures [21], that target screening for women age 50–74. The USPSTF recommendations were associated with decreased screening among women in age groups for whom recommendations either did not apply (younger than 40); were changed from universal to selective (40–49); or the evidence of effectiveness was lacking (75 and older). The recommendation may have discouraged screening among women younger than 40 who were never appropriately included in the screening pool unless they were at high-risk for breast cancer (e.g., genetic mutation, previous high risk breast lesion). Although many women continued screening after the new recommendations were issued, our study was unable to determine the rationale and decision making process for screening in these women. Implementation of the Affordable Care Act was associated with increased screening among women age 50–74, reinforcing screening efforts in this population. It is possible that economic barriers to screening, whether real or perceived, may have been diminished by insurance coverage expansion. Screening in other age groups was not affected by the Affordable Care Act.

The effects of the USPSTF recommendations and Affordable Care Act on women with different types of insurance were mixed. The USPSTF recommendations were associated with decreased screening among women with commercial insurance, while the Affordable Care Act was associated with increased screening for women with Medicare, Medicaid, or other noncommercial sources of payment. Since age and insurance type are significantly related (*P*<0.001), their relationships with screening were confounded.

Our results are inconsistent with other published studies of the impact of the USPSTF recommendations [9–12]. Two smaller studies based on self-reported information indicated no statistically significant differences in mammography rates over time for all age groups studied [9, 10]. These include a study of 29,857 women responding to Medical Expenditure Panel Surveys from 2006 to 2010 [9], and a study of 27,829 women age 40 and older from the National Health Interview Survey from 2008 to 2011 [10]. In contrast, two large studies indicated statistically significant declines in mammography [11, 12]. These include a retrospective study of a statewide mammography registry of 150,000 women age 40 and older from 2009 to 2011 [11], and an analysis of 5.5 million insured women age 40–64 from 2005 to 2013 [12]. None of these studies consider other influences on screening rates besides the USPSTF recommendations. They also differ by using self-reported or registry data, comparing screening rates from one time period with another, using shorter or dissimilar time periods, or enrolling varying numbers of participants.

A recently published time-series analysis of several million privately insured women obtaining screening mammography between 2006 and 2011 reported a decline in screening rates two months after release of the USPSTF recommendation among women age 40–49, but not women age 50–64 [15]. However, this decline was not sustained after two years, contrasting with our results that indicated a sustained decline in screening for age 40–49. Results of both studies showed no significant declines for women age 50–64 for whom screening recommendations did not change. Differences in results for women age 40–49 could be related to methodological differences between the studies, particularly the inclusion of only privately insured women compared with all types of payers; inclusion of data from different time periods; use of interrupted time-series analysis versus intervention analysis for time-series data; including screening and diagnostic mammograms compared with screening mammograms only; and assumptions of expected baseline screening rates. Importantly, our study also considered the concurrent effect of implementation of the Affordable Care Act that exhibited its own effect on screening.

The strengths of this study include its use of patient-level data from a large health system that closely represents the diversity of patients, providers, and practices across a broad geographic region, improving its applicability. In addition, we used highly reliable data from actual screening mammography, rather than self-reported accounts, from nearly 250,000 encounters over a sufficiently long period of time to appropriately evaluate changes and interventions. We used a highly accurate method of identifying and integrating patient data across the health system assuring completeness and accuracy.

This study may be limited by its use of data for women actually screened without considering the pool of candidates that was not screened, as would be available in a population-based registry or closed health system. Patients enter and leave the health system and it is possible that specific subgroups preferentially migrated, such as those of a certain age or insurance type. However, major demographic shifts of patients obtaining care at the health system for other health services have not been observed. Patient migration could potentially occur in closed systems and registries over time as well. Also, we used an analytic approach that was appropriate for our data and was not dependent on screening rates. Health systems often require pragmatic approaches to analyzing and interpreting data based on electronic health system data sources (i.e., big data) such as we used in this study.

The analysis was based on several assumptions. Our approach assumed that other than the specified interventions, all other factors that might affect the screening volume (the underlying intrinsic fluctuation of the process) stayed the same over the study period. It is possible that other factors could have had important influences. Also, we assigned a specific time point for implementation of the Affordable Care Act, when there was actually a phasing in period. However, our rationale for using January 2011 as the starting point was based on the implementation of insurance plans in our health system and the simplicity and ease of interpretation of the model. We assumed that intervention effects were step changes, even though changes in medical practice are often gradual. To test this assumption, we modified the modeling process with a ramping up effect and the results did not indicate differences in the model. Although these findings support our assumptions, they could also reflect the limited number of time points in the time series rather than the absence of effect. In addition, our assumption that the interventions only affected the mean mammography volume may not be correct if the intervention also changed the intrinsic fluctuation (e.g., by altering the variance of the background noise). Due to the small number of time points in the data, the validity of this assumption could not be meaningfully assessed.

Future studies of screening changes in other health systems could provide additional information about the impact of changes in practice recommendations and insurance coverage, as well as other factors. Results of these studies would be particularly useful to health systems providing screening services.

In conclusion, mammography screening volumes in a large community health system decreased among women younger than 50 and 75 and older in association with new USPSTF practice recommendations, while insurance coverage changes under the Affordable Care Act were associated with increased screening volumes among women age 50–74. These changes align with current practice standards and quality performance measures that target screening for women age 50–74.
